# Clinical characteristics analysis of pediatric spinal cord injury without radiological abnormality in China: a retrospective study

**DOI:** 10.1186/s12887-024-04716-z

**Published:** 2024-04-03

**Authors:** Renfeng Liu, Qizhi Fan, Jingpeng He, Xin Wu, Wei Tan, Zuyun Yan, Weiguo Wang, Zhiyue Li, You-Wen Deng

**Affiliations:** Department of Spinal Surgery, The Third Xiangya Hospital of Central South University, Changsha, Hunan Province China

**Keywords:** SCIWORA, Chinese children, Sports-related, Dancing, Backbend, Pediatric

## Abstract

**Purpose:**

This study aims to analyze the clinical characteristics of Chinese children with spinal cord injury (SCI) without radiographic abnormality (SCIWORA) and explore their contributing factors and mechanisms of occurrence.

**Methods:**

A retrospective analysis was conducted on the clinical data of pediatric patients diagnosed with SCIWORA from January 2005 to May 2020. Epidemiological, etiological, mechanistic, therapeutic, and outcome aspects were analyzed.

**Results:**

A total of 47 patients with SCIWORA were included in this study, comprising 16 males and 31 females. The age range was 4 to 12 years, with an average age of 7.49 ± 2.04 years, and 70% of the patients were below eight. Sports-related injuries constituted 66%, with 70% attributed to dance backbend practice. Thoracic segment injuries accounted for 77%. In the American Spinal Injury Association (ASIA) classification, the combined proportion of A and B grades accounted for 88%. Conservative treatment was chosen by 98% of the patients, with muscle atrophy, spinal scoliosis, hip joint abnormalities, and urinary system infections being the most common complications.

**Conclusion:**

SCIWORA in Chinese children is more prevalent in those under eight years old, with a higher incidence in females than males. Thoracic spinal cord injuries are predominant, dance backbend as a primary contributing factor, and the social environment of “neijuan” is a critical potential inducing factor. Furthermore, the initial severity of the injury plays a decisive role in determining the prognosis of SCIWORA.

## Background

Spinal Cord Injury (SCI) Without Radiographic Abnormality (SCIWORA), first described in 1974 [1], was subsequently defined by Pang and Wilberger in 1982 as “SCI without evidence of vertebral fracture or dislocation on X-ray and computed tomography (CT), excluding penetrating injuries, electrical injuries, birth trauma, and congenital spinal deformities [2].” SCIWORA accounts for 13–42% of all pediatric spinal cord injuries, posing a high risk of disability due to the lack of effective treatment methods [3, 4]. Children’s specific physiological and anatomical characteristics are considered the basis for SCIWORA. Compared to adults, children have a larger head-to-trunk ratio, relatively weaker neck muscles, vertically oriented vertebral bodies, and higher water content in intervertebral discs and vertebral rings. From an anatomical perspective, children are more prone to this specific type of SCI [3, 5, 6].

Many hospitals and institutions in Western countries have researched pediatric SCIWORA (PSCIWORA), summarizing their experiences in diagnosis, treatment, and outcomes [3, 5, 7, 8]. With China’s large population, the number of PSCIWORA patients is higher than in other regions. Unfortunately, there are few related research reports in China, and based on the cases we have encountered, the characteristics of Chinese patients differ from those reported in Western countries. Therefore, this retrospective case study aims to analyze and summarize the clinical features of PSCIWORA in China.

## Methods

### Patients

Clinical data of children diagnosed with SCIWORA admitted to our hospital from January 2005 to May 2020 was collected, including age, gender, cause of injury, location of injury, diagnostic imaging methods, treatment strategies, neurological function grading, and prognosis. Analysis was performed on the etiology, incubation period, diagnosis, lesion level, treatment, and outcomes. Inclusion criteria: (1) Age < 18 years; (2) Clinical symptoms and signs of SCI; (3) There is no evidence of spinal column fracture or dislocation on X-ray and CT. Exclusion criteria: (1) penetrating injuries, electrical injuries, or birth trauma; (2) history of neurological or psychiatric diseases; (3) concurrent traumatic brain injury; (4) congenital spinal deformities. This retrospective study followed institutional and national research committee ethics, and approval was obtained from the ethics committee (Ethics No. Fast 23,805).It also has been registered in the Clinical Trial Registry, retrospectively registered.

### Diagnosis

Upon emergency admission, all patients underwent a neurological examination to assess their motor and sensory functions. The American Spinal Injury Association (ASIA) classification evaluated the neurological status. X-rays and CT were used to assess fractures, subluxations, dislocations, deformities, and soft tissue injuries. Conventional magnetic resonance imaging (MRI) was further employed to assess spinal cord and soft tissue injuries. Further evaluation was conducted for patients with neurological symptoms and negative findings on routine MRI using MR diffusion tensor imaging (DTI).

### Treatment

Patients diagnosed with SCIWORA were typically advised bed rest and immobilization. They may be required to wear appropriate braces to maintain spinal stability and prevent secondary injuries. Additionally, bed rehabilitation training includes turning, resetting, massage, and exercise therapy. Catheterization was performed for patients with urinary retention. For patients with clinical symptoms within three days, methylprednisolone or dexamethasone is typically administered for shock treatment. Mannitol was used to eliminate spinal cord and soft tissue edema. Additionally, neurotrophic drugs such as mecobalamin and ganglioside were added for auxiliary treatment. Vasodilators were administered to improve spinal cord blood microcirculation after ruling out hemorrhagic diseases. Patients were generally advised to undergo neurorehabilitation treatment and hyperbaric oxygen therapy in a rehabilitation hospital once their condition stabilized.

### Statistical analysis

Utilize the Statistical Package for the Social Sciences (SPSS) version 24.0 (IBM, Armonk, New York, USA) and Microsoft Excel (Microsoft Corporation, Redmond, Washington, USA) to manage and analyze data. Calculate descriptive statistics for SCIWORA patients, primarily employing frequency analysis. Individual characteristics were described as numerical values or mean ± standard deviation.

## Results

### Epidemiology

A total of 47 cases of PSCIWORA were collected, with 16 males and 31 females, resulting in a male-to-female ratio close to 1:2. The average age was 7.49 ± 2.04 years, and the majority were under eight years old, accounting for 70% (Table 1).

**Table 1:** Demographics, treatments, and complications

		Data	Percentage (%)
**People counting**			
	Headcount	47	
	< 8 years old	33	70
	8–18 years old	14	30
**Gender**			
	Male	16	34
	Female	31	66
**Age** Mean (SD)			
	Total	7.49 (2.04)	
	Male	8.25 (1.91)	
	Female	7.10 (2.02)	
**Incubation period**			
	0–1 h	41	88
	1–4 h	3	6
	4–24 h	3	6
**Therapy**			
	Surgery	1	2
	Conservative	46	98
**Complication**			
	Muscular atrophy	46	98
	Scoliosis	32	68
	Dysplasia of the hip	26	55
	Urinary tract infection	20	43
	Hydronephrosis	7	15
	Dermatitis	5	10
	Urethral calculi	4	9
	Pressure sores	3	6
	Fragility fractures	2	4

### Etiology of injury

Sports-related trauma accounted for the majority of injuries (66%), with traffic accidents being the second most common cause (19%). Violence, falls, and other causes accounted for 9%, 4%, and 2% of injuries, respectively. Dance backbend practices accounted for 71% of all sports-related injuries (Table 2).

**Table 2:** Injury characteristics and MRI detection

			Data	Percentage (%)
**Cause of injury**	Sports		31	66
		Backbend	22	71
		Somersault	6	19
		Ball game	3	10
	Traffic accident		9	19
	Fall off		4	9
	Violence		2	4
	Other		1	2
**Level of lesion**	Cervical		9	19
	Cervicothoracic		6	13
	Thoracic		22	47
	Thoracolumbar		8	17
	Lumbar		2	4
**MRI**	Positive		43	91
	Negative		4	9

### Location of injury

**Fig. 1:**
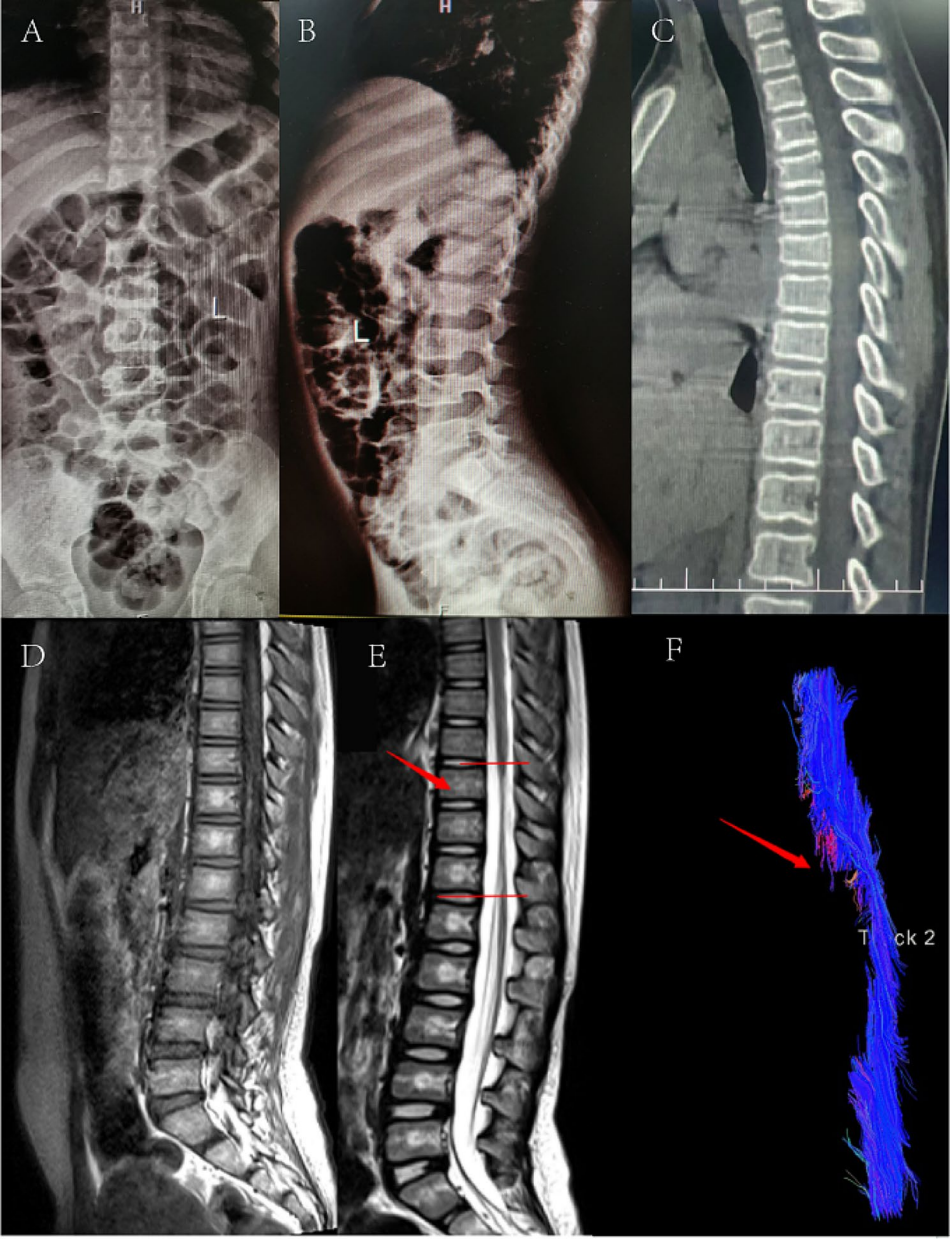
A 10-year-old female patient presented with paralysis after dance backbend practice. X-ray (**A, B**) and CT (**C**) scans did not detect any fractures or dislocations. No abnormmality was found on T1-weighted MRI (**D**), T2-weighted MRI (**E**) showed localized spinal cord thinning at the T10-12 vertebral level. DTI fiber reconstruction images (**F**) revealed local fiber areas and thinning of fiber bundles

**Fig. 2:**
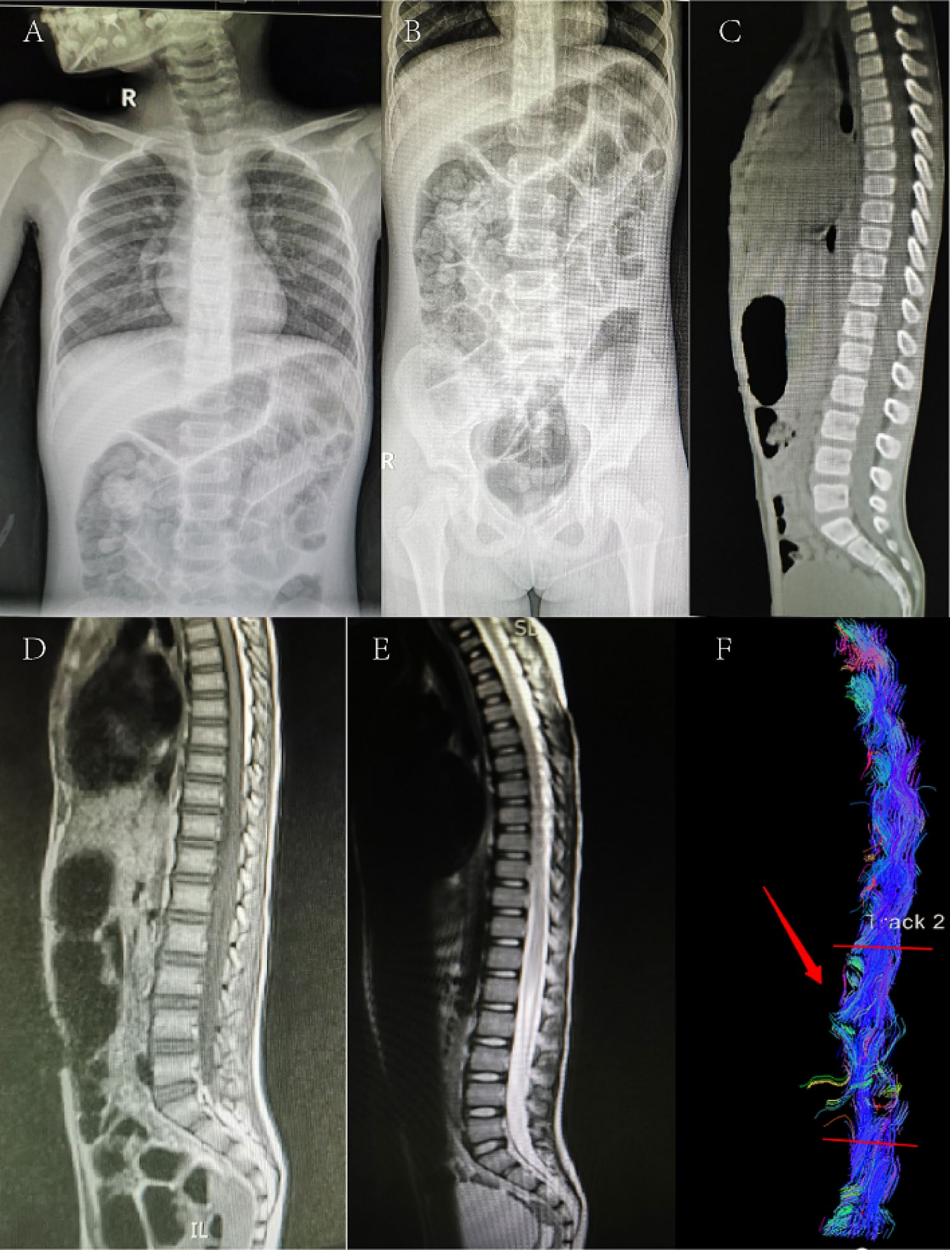
An 8-year-old female patient presented with paralysis after dance backbend practice. X-ray (**A, B**) and CT (**C**) scans did not detect any fractures or dislocations. MRI (**D, E**) examination also showed no apparent abnormalities. However, DTI fiber reconstruction images (**F**) revealed localized thinning, fracture, and sparse fibers below the ninth thoracic vertebra

Through routine MRI examinations, injury sites were identified in 91% of patients. In a small subset of patients with negative findings on conventional MRI, DTI revealed the disruption of nerve fibers (Figs. 1 and 2). The majority of spinal cord injuries involved the thoracic segment, with proportions of 13%, 47%, and 17% for the cervical-thoracic, thoracic, and thoracolumbar regions, respectively. 19% of spinal cord injuries were cervical, while 4% were lumbar. (Table 2).

### Treatment and outcome

Most patients exhibited severe neurological dysfunction immediately after injury, with 28 cases of ASIA classified as grade A and 13 cases as grade B, accounting for a total proportion of 88% (Table 3). Most patients (98%) opted for conservative treatment due to the inability to identify specific spinal cord compressions. At the last follow-up, only one case of grade D improved to normal after surgical treatment, while the others still had neurological dysfunction. Three cases improved from grade A to B, one case improved from grade A to C, four cases improved from grade B to C, and two cases improved from grade C to D (Table 3). Various complications were observed in the follow-up, with muscle atrophy, spinal scoliosis, hip joint abnormalities, and urinary system infections being the most common. Some patients developed complications such as pressure sores, dermatitis, urethral stones, renal pelvis dilation, and brittle bone fractures due to poor care (Table 1).

**Table 3:** ASIA classification of spinal cord injury

	A	B	C	D	E
**First Consult**	28	13	5	1	0
**Percentage (%)**	60	28	10	2	0
**The final follow-up**	24	12	8	2	1
**Percentage (%)**	51	26	17	4	2

## Discussion

SCIWORA is a term commonly used to describe a specific type of pediatric SCI, with 90% of SCIWORA cases occurring in children [4]. During childhood, a particular physiological developmental stage, a combination of a relatively larger head, shallow-angled small joints, lax ligaments, and immature Luschka joints, results in toddlers having a hypermobile spine with a pivot point for movement different from that of adults. The range of motion in the spine is significantly greater than that of spinal cord activity [7]. When the body undergoes excessive flexion or extension along the longitudinal axis, the spinal cord and meninges cannot adapt to the substantial elastic changes in the spine, leading to traction or compression injuries to the spinal cord. Additionally, In children under eight, the sagittal and transverse diameters of the spinal canal are narrowest at the level of T4–9, the anastomosis between the extraspinal and intraspinal arteries is minimal, making the spinal cord vulnerable to even slight traction or flexion, which can result in ischemic necrosis [9]. As age increases, spinal stability improves, enhancing tolerance to low-energy injuries [10]. Therefore, SCIWORA is more prevalent in children under eight years old, consistent with our epidemiological observations.

Research has indicated that due to the instability of the cervical spine in young children, pediatric SCIWORA often occurs in the cervical region, primarily manifesting as incomplete SCI [11]. Dynamic imaging studies have shown that the pivot point of the cervical spine in maximal flexion shifts from C2-C3 in infants and toddlers to C3-C4 at 5–6 years old, reaching C5–C6 in adulthood [12]. Consequently, in children under eight years old, injuries are more likely to occur in the upper cervical segments. However, beyond the age of 8, the anatomical structure of the pediatric cervical spine approaches that of adults, with increased stability in the upper cervical spine and a subsequent rise in the incidence of injuries to the lower cervical spine. These findings suggest that the upper cervical segments have the greatest physiological mobility during infancy and early childhood but are also the most susceptible to flexion injuries [9]. Surprisingly, the thoracic spine was predominant in our study and most of the patients presented with complete spinal cord injuries. These discrepancies may be attributed to differences in the patients’ mechanisms and causes of injuries. According to previous reports, the top three causes of pediatric traumatic spinal cord injuries in regions such as East Asia, North America, and Oceania are traffic accidents (46–74%), falls from heights (12–35%), and sports and entertainment activities (10–25%) [13]. Traffic accidents are often associated with whiplash injuries, with the cervical spine being the most commonly affected area [14]. However, in our study, the primary cause of injuries was sports-related (66%), with 71% of sports-related injuries occurring in the lower back. According to Wang et al.‘s study [15], there are similar discrepancies between Chinese and foreign studies, but they did not explore the potential causes of these differences. We believe that the societal phenomenon of “Neijuan” in Chinese society may contribute to this variability.

“Neijuan” is a popular Chinese internet term that refers to the phenomenon where individuals excessively strive for success in an intensely competitive social environment, leading to an unhealthy competitive atmosphere and social phenomenon. This phenomenon is prevalent in China. Influenced by prolonged exposure to this unhealthy competitive atmosphere, parents project their societal anxiety onto the care of the next generation, often articulated as “Do not let the children fall behind at the starting line.” Consequently, Chinese children have to face numerous additional extracurricular classes, and dance is one of the primary choices. The foundational elements of dance focus on enhancing muscle flexibility, with bending at the waist a crucial movement. Due to the developmental stage of a child’s spine, characterized by a lax and unstable anatomical structure, the spine’s adaptability is greater than that of the spinal cord [10]. When a child bends at the waist, most stress concentrates on the mid-thoracic vertebrae, leading to extreme thoracic spine extension [16]. Nevertheless, the cervical vertebrae, lumbar vertebrae, medulla oblongata, and conus cauda equina are relatively fixed in position. Passive stretching of the anterior longitudinal ligament and the protrusion of the wrinkled yellow ligament into the vertebral canal further reduce the reserve space within the vertebral canal [16]. When the stress level reaches a certain level, the facet joints may immediately experience horizontal sliding or vertebral dislocation. This puts the spinal cord under a huge amount of stress at once, which can cause both transverse and complete spinal cord injuries [8]. Simultaneously, due to poor blood supply to the spinal cord in the T2 to T4 segments, the incidence of thoracic segment SCIWORA is relatively high. Some also believe that it may be related to fibrocartilaginous embolism [17]. Bending at the waist can damage the intervertebral discs and the blood vessels still present in the intervertebral discs of young children, causing mucoid fibrous cartilaginous emboli to enter the central artery of the spinal cord, leading to spinal cord ischemia.

The primary manifestations of PSCIWORA injuries include spinal cord contusion, compression, and traction. Early identification of these injuries is crucial for prompt treatment and minimizing the degree of neurological deficit. Traditional radiological examinations, including X-ray and CT scans, are necessary for appropriately assessing the extent and precise location of the injury. However, the challenge arises when X-ray and CT imaging results are negative and biochemical tests such as cerebrospinal fluid analysis and blood routine show no positive findings, making the accurate diagnosis of SCIWORA challenging [18]. The application of MRI provides extensive information on neural and soft tissue injuries [19]. In cases of SCI, MRI can reveal bleeding, ischemia, or swelling with high-intensity signals on T2-weighted images [20, 21]. Despite the powerful capability of MRI in detecting soft tissue injuries, some spinal cord injuries remain undetectable through conventional MRI scans. In our study, most patients’ injuries were precisely located through routine MRI. In contrast, four patients showed no abnormalities on magnetic resonance but were diagnosed through further diffusion tensor imaging (DTI). DTI is a technique based on diffusion-weighted imaging (DWI) that applies a gradient field with 6–55 nonlinear directions to obtain a diffusion tensor image, quantitatively depicting the three-dimensional trajectory of water molecule diffusion in space [22]. The structural morphology of nerve fiber bundles can be visualized through DTI reconstruction and post-processing fiber tracking (FT) techniques. Currently, DTI and FT technologies are widely used in the nervous system. Studies have shown that some patients without conventional MRI abnormalities were found to have high-density signal abnormalities using DTI examinations [23, 24]. In this study, using DTI technology, we saw fiber discontinuity in the spinal cord of four patients whose regular MRI results were negative. This helped us figure out where the injury was. Therefore, for SCIWORA patients exhibiting persistent neurological symptoms without MRI abnormalities, we recommend utilizing DTI examinations to enhance MRI diagnostic sensitivity and explore potential subtle spinal cord injuries.

Given the absence of evident signs of spinal trauma, such as fractures and dislocations, in SCIWORA patients, conservative treatment is the primary therapeutic approach [25]. Conservative treatment involves strict bed rest, immobilization, early administration of high-dose methylprednisolone, dehydration agents, neurotrophic drugs, and hyperbaric oxygen therapy [10]. The primary goal is to protect the remaining gray matter function of the spinal cord and prevent white matter necrosis, maximizing the recovery of spinal cord function. Vasoconstriction and ischemia of spinal cord vessels are the main contributors to PSCIWORA [26]. Consequently, pharmacological interventions should prioritize drugs that alleviate vascular spasms, dilate blood vessels, prevent microthrombus formation, and increase spinal cord blood flow. Dehydration, steroids, and neurotrophic drugs are also part of the treatment regimen. Although a guideline from 2002 recommended a 24-hour course of steroid pulse therapy within 8 h of SCI, there is still controversy surrounding its efficacy [27]. In our study, most PSCIWORA patients showed signs of neurological dysfunction within one hour of injury. Although steroid pulse therapy was administered, only a tiny fraction exhibited improved neurological function, with most patients showing no change in function before and after treatment. Consequently, considering the critical evaluation of the effectiveness and safety of steroid treatment and the existing guidelines, clinicians should exercise caution when considering steroid pulse therapy in the treatment of spinal cord injuries. Moreover, research indicates that early combined hyperbaric oxygen therapy can effectively alleviate spinal cord edema, increase oxygen pressure and blood oxygen content, enhance blood oxygen diffusion radius, improve the ischemic and hypoxic states of the spinal cord, reduce neuronal apoptosis, promote nerve regeneration and repair [28, 29]. In our study, all patients were recommended for subsequent hyperbaric oxygen therapy, but the neurological recovery outcomes were minimal. This may be correlated with the initial state of neurological damage, as most patients in our study presented with initial neurological function in ASIA grades A and B. Therefore, it is crucial to recognize the determining role of the initial injury severity in treatment outcomes and prognosis [10].

The complexity of treating neural injuries results in a less favorable prognosis for a considerable portion of individuals with SCIWORA, which subsequently leads to the manifestation of diverse long-term consequences. Our study found that musculoskeletal and urinary system difficulties were the prevailing issues in PSCIWORA, which aligns with prior findings [30]. Neural dysfunction leads to limited movement of the limbs, which causes muscles to undergo disuse atrophy. Moreover, spinal cord injuries impact the growth and maturation of the skeletal system, and prolonged periods of lying down or sitting in patients can lead to scoliosis and abnormal development of the hip joints [31]. Enhancing rehabilitation training, engaging in passive muscle exercises, and maintaining appropriate posture are effective methods for preventing spine curvature and improper hip joint development. Spinal cord damage frequently leads to impaired urine function, necessitating extended use of catheters and heightening susceptibility to urinary tract infections and bladder stones [32]. Early implementation of intermittent catheterization is a dependable approach for reducing difficulties in the urinary system. Timely, comprehensive rehabilitation treatment can significantly reduce the disability rate of PSCIWORA, promote functional recovery, and improve the quality of life [30].

### Limitation

This study has several limitations. Firstly, PSCIWORA is uncommon, and China lacks a systematic nationwide medical record database. As a result, the number of cases collected in this study is relatively small, and it is a single-center study, potentially introducing bias. Secondly, there needs to be more comparative studies with other types of spinal cord injuries, preventing a complete elucidation of the unique characteristics of SCIWORA. Therefore, subsequent multicenter studies with a larger sample size and a case-control design are needed to address these limitations.

## Conclusion

SCIWORA in Chinese children is more prevalent in those under eight years old, with a higher incidence in females than males. Thoracic spinal cord injuries are predominant, dance backbend as a primary contributing factor, and the social environment of “neijuan” is a critical potential inducing factor. Furthermore, the initial severity of the injury plays a decisive role in determining the prognosis of SCIWORA.

## Data Availability

The dataset analyzed during the current study is not publicly available but can be obtained from the corresponding author upon reasonable request.

## Declarations

**Acknowledgment**.

## Abbreviations

ASIA: American Spinal Injury Association
CT: Computed tomography
DTI: diffusion tensor imaging
DWI: Diffusion-weighted imaging
FT: Fiber tracking
MRI: Magnetic resonance imaging
PSCIWORA: Pediatric spinal cord injury without radiological abnormality
SCI: Spinal cord injury
SCIWORA: Spinal cord injury without radiological abnormality
